# Bullous pemphigoid induced by anti-IL-23 monoclonal antibody in a psoriatic patient: a case report

**DOI:** 10.3389/fmed.2025.1709423

**Published:** 2025-12-18

**Authors:** Rong Xi, Yi Cao, Qian Zhuang, Lili Ma, Maocan Tao, Yuanyuan Li

**Affiliations:** The First Affiliated Hospital of Zhejiang Chinese Medical University (Zhejiang Provincial Hospital of Chinese Medicine), Hangzhou, China

**Keywords:** autoimmune disease, biologics, drug reaction, immunobullous disease, psoriasis

## Abstract

Psoriasis, a chronic immune-mediated inflammatory skin disorder, is increasingly managed with anti-IL-23 biologics, such as guselkumab. Nevertheless, their rare adverse effects remain poorly understood. This study presents the case of a 78-year-old woman with moderate-to-severe psoriasis who presented with bullous pemphigoid (BP) 2 weeks after initiating guselkumab therapy. The clinical presentation was characterized by generalized bullae with epidermal–dermal separation and eosinophilic infiltration, despite negative anti-BP180 or BP230 antibodies. Single-cell RNA sequencing (scRNA-seq) of psoriatic versus BP lesions exhibited distinct cellular profiles: BP lesions were enriched in epidermal stem cells (44.32%) and endothelial cells (21.38%), in contrast to keratinocyte predominance (77.3%) noted in psoriasis. Differential gene analysis revealed the upregulation of keratinocyte stress markers (KRT6B and MMP7) and interferon-related genes (IFI6) in BP. Immune dysregulation was reflected in the activation of macrophages/T cells expressing pro-inflammatory factors (SPP1 and GZMB). Tissue stem cells demonstrated PTX3 upregulation, linking complement activation and extracellular matrix remodeling to epidermal damage. Collectively, these findings reveal dual mechanisms of guselkumab-induced BP: immune imbalance and defective epidermal repair. Future studies should validate these pathways in larger cohorts and investigate IL-23/interferon signaling crosstalk.

## 1 Introduction

Psoriasis is a chronic immune-mediated dermatosis affecting approximately 125 million individuals worldwide, with higher prevalence noted among adults, populations in high-income countries, and older adults (1). For the past two decades, biologics have refined the treatment of moderate-to-severe plaque psoriasis through their efficacy and favorable safety profiles. These agents effectively target specific molecular pathways involved in disease pathophysiology (2). Biologic therapies are currently regarded as cornerstone treatments for moderate-to-severe psoriasis, owing to their proven effectiveness and safety. Some of the commonly encountered adverse drug reactions are generally mild, most often manifesting as influenza-like symptoms or localized cutaneous effects such as injection-site reactions. Drug-induced bullous pemphigoid (DBP), a rare phenomenon, has been observed with certain biologics, particularly anti-TNF-*α* agents and anti-IL-12/IL-23 drugs such as ustekinumab—with only limited case reports associated with guselkumab (anti-IL-23) (3).

Although, recent case reports have documented DBP following biologic therapy, the mechanisms underlying guselkumab-associated DBP remain poorly understood. High-resolution, cell type-specific transcriptomic analyses are critical for understanding cellular functions. The scRNA-seq enables effective quantification of population heterogeneity and investigation of cellular states and transitions at unprecedented resolution, revealing cell subtypes or gene expression patterns that are often masked by bulk analyses (4). In this study, we conducted a parallel comparison of transcriptomic profiles between psoriatic and DBP lesions from the same patient and identified lesion-specific differentially expressed genes (DEGs). Using unbiased scRNA-seq of concurrently obtained lesions, cell type-specific transcriptomic differences between these two distinct pathological states were analyzed and delineated. Furthermore, written informed consent was obtained from the patient both for case details and image publication.

This study presents the case of a 78-year-old woman with a history of moderate-to-severe psoriasis who developed generalized BP 2 weeks after initiating guselkumab therapy. The diagnosis of drug-induced BP was confirmed by histopathological tests and a strong temporal association with drug administration, despite seronegativity for anti-BP180 and anti-BP230 antibodies. The adverse effects resolved completely following discontinuation of guselkumab and initiation of systemic corticosteroid therapy. To evaluate the underlying mechanisms, we performed a comparative single-cell transcriptomic analysis of psoriatic and BP lesions from the same patient, revealing distinctive cellular landscapes and pathogenic pathways associated with this paradoxical reaction.

## 2 Case presentation

**Figure 1:**
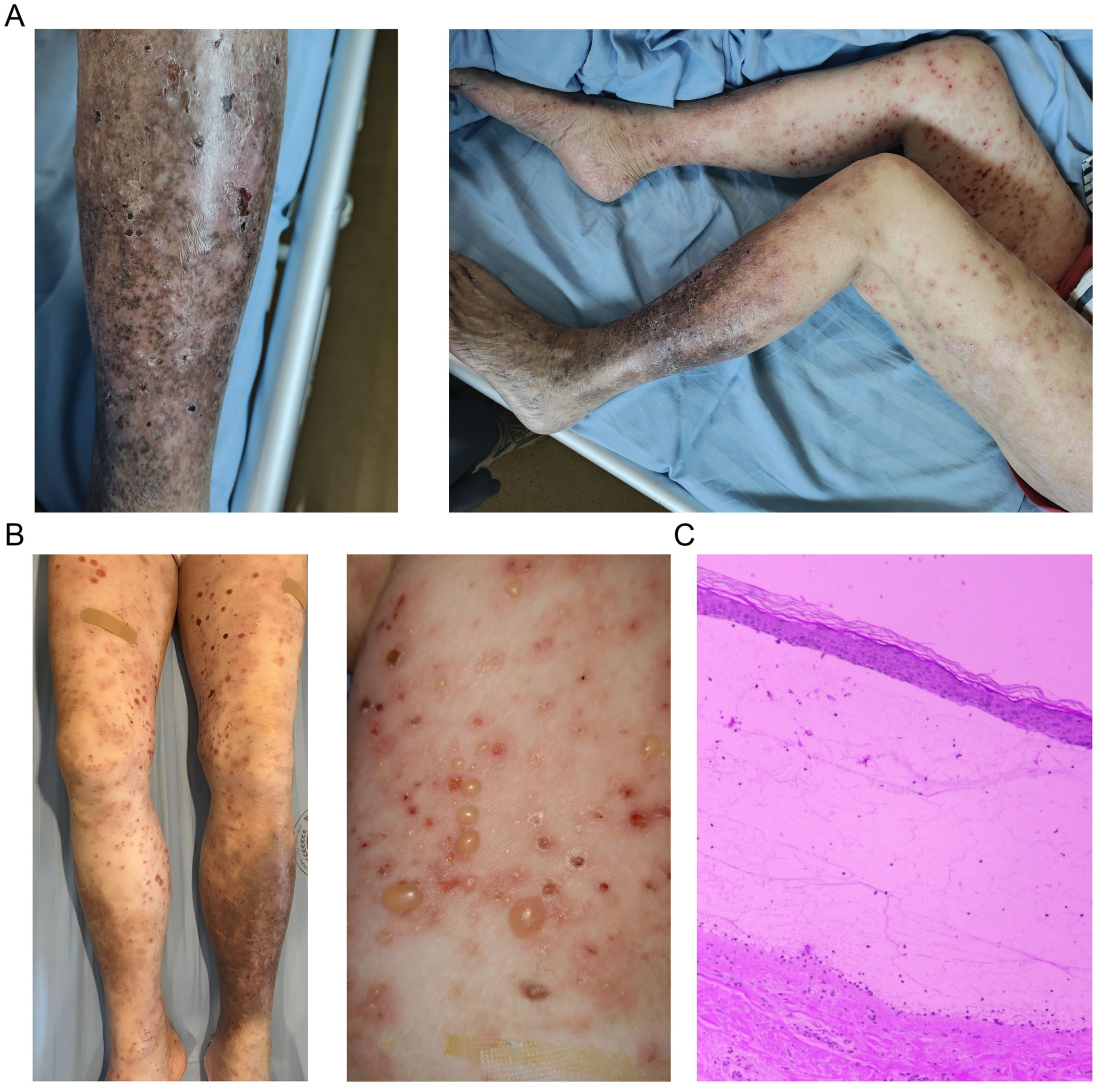
Clinical and histopathological manifestations of the rash. **(A)** Representative psoriatic plaque lesions on the lower extremities before biologic intervention, showing characteristic erythematous, well-demarcated plaques with silvery scales. **(B)** Generalized bullous eruption emerging 2 days after the first guselkumab injection, demonstrating tense bullae on erythematous bases distributed across the trunk and proximal extremities. **(C)** Histopathological examination of a bullous lesion confirming the diagnosis of bullous pemphigoid, featuring a subepidermal split with a prominent inflammatory infiltrate rich in eosinophils (black arrows) in the superficial dermis (H&E staining, 200 × magnification).

We present the case of a 78-year-old woman with a history of moderate-to-severe plaque psoriasis (PASI 20), characterized by well-demarcated erythematous scaly plaques on the trunk and extremities (Figure 1A), who presented with generalized tense bullae on erythematous-edematous lower limbs. The patient received a single subcutaneous dose of guselkumab (100 mg) on 9 July 2024. The bullous eruption appeared within 2 days of the initial injection, demonstrating an exceptionally rapid onset after the initial drug exposure.

The patient’s medical history included hypertension for over 30 years, type 2 diabetes mellitus for over 30 years, and hyperlipidemia for more than 10 years. Her medication regimen remained stable for more than 1 year prior to the onset of the bullous eruption and included valsartan/amlodipine for hypertension, along with acarbose, sitagliptin/metformin, and dapagliflozin for diabetes, together with aspirin, atorvastatin, and ezetimibe for hyperlipidemia. Given the extended stability of this treatment regimen well before the appearance of bullous lesions, these medications were considered unlikely etiologic factors for the pemphigoid eruption. No additional medications were introduced, and no dosage adjustments were made in the chronic therapies for a period of 6 months preceding the onset of bullous lesions. Other comorbidities included essential hypertension, controlled with amlodipine 5 mg daily. Physical examination showed widespread tense bullae on erythematous and edematous skin, spread around an area of approximately 30% of the body surface (Figure 1B). Lesions were scattered all over the trunk and proximal extremities, with significant involvement of flexural regions. The tense bullae, measuring 1–4 cm in diameter, arose on both erythematous and non-inflamed skin. A complete examination of the oral, genital, and ocular mucosa was performed. Notably, no vesicles, erosions, or other signs of mucosal involvement were observed. A skin biopsy for histopathological analysis was performed on 29 July 2024, approximately 3 weeks after the initial guselkumab injection and the subsequent development of bullous lesions. Histopathological examination of bullous lesions revealed a subepidermal cleavage with prominent eosinophilic infiltration (Figure 1C). Commercially available enzyme-linked immunosorbent assay (ELISA) systems (MBL, Nagoya, Japan), which specifically target the immunodominant NC16A domain of BP180 and the C-terminal region of BP230, exhibited negative results for circulating autoantibodies, thereby supporting the diagnosis of DBP. Direct immunofluorescence was not performed, as the clinical presentation, characteristic histopathological findings, and the strong temporal association with drug initiation were collectively deemed sufficient for a working diagnosis of DBP. The Naranjo Adverse Drug Reaction Probability Scale score was calculated as 7, indicating a “probable” relationship between guselkumab exposure and the adverse events (5). Guselkumab was immediately discontinued. The patient was subsequently treated with intravenous methylprednisolone (40 mg/day), which led to rapid clinical improvement. Complete resolution of the lesions was observed within 1 month, with no recurrence during the follow-up period while the patient was maintained on a tapering dose of prednisone.

### 2.1 Overview and identification of main cell types in psoriatic and pemphigoid tissues using scRNA-seq

Patients were included in the experimental research after providing signed consent forms preoperatively. Baseline data, including gender, age, imaging results, and pathological information, were collected prior to surgery. Then, samples were taken from distinct psoriatic plaques (Supplementary Figure S1A) and also from the newly formed blister lesions (Supplementary Figure S1B). A whole blister was excised from the blister site with an incision approximately 5 mm in diameter, reaching the fat layer. After excision, the tissue was immediately rinsed in sterile saline, stored in tissue preservation solution, and sent to Hangzhou Luca Biotechnology Co., Ltd. (Hangzhou, China) for further examination.

Single-cell suspensions were prepared for sequencing. Skin samples were inoculated in a dissociation solution (composition specified as PBS containing 0.25% trypsin and 1 mg/mL collagenase) and gently shaken at 100 rpm in a 37 °C water bath for 20 min. Digestion was terminated by adding PBS with 10% fetal bovine serum (FBS, v/v). Cells were resuspended in PBS and incubated with 10-fold diluted red blood cell lysis buffer (MACS 130-094-183). After incubation, the cells were centrifuged at 300 × g for 5 min at 25 °C, and the dead cells were removed using the Miltenyi^®^ Dead Cell Removal Kit (MACS 130-090-101). Finally, the separated cells were resuspended in PBS, viability was assessed by Trypan blue exclusion, and cells were counted using a hemocytometer or Countess II automated cell counter. Total cell viability was >85%, and the final cell concentration was adjusted to 700–1,200 cells/μL.

Following the manufacturer’s instructions, the 10 × Genomics Chromium Single-Cell 3’ Reagent Kit (V3) was used to capture approximately 5,000 single cells (6). RNA sequencing libraries were then constructed according to standard protocols. Libraries were sequenced on the Illumina NovaSeq 6,000 sequencing system (paired-end, 150 bp), with a minimum sequencing depth of 20,000 reads per cell.

The Cell Ranger pipeline (version 4.0.0) was used for sample demultiplexing, barcode processing, single-cell 3′ gene expression counting, and genome alignment (GRCh38, v96).

Furthermore, the Seurat R package (version 3.1.1) was used for quality control. Genes expressed in fewer than three cells or with unique molecular identifier (UMI) counts less than 500 were removed, and cells with mitochondrial gene expression percentage greater than 25% were also excluded. The Seurat R package was also used for data filtering, normalization, principal component analysis (PCA), and t-distributed stochastic neighbor embedding (t-SNE), with thresholds as reported in a previous study (7).

The Seurat R package was used for analyzing DEGs in different cell populations via a bimodal likelihood ratio test and identifying genes upregulated in more than 10% of cells in different populations with a *p*-value of ≤ 0.01 and log2 fold change [log_2_*fold change* (*FC*)] ≥ 0.25. Gene ontology (GO; http://geneontology.org/) and Kyoto encyclopedia of genes and genomes (KEGG; www.genome.jp/kegg) analyses were carried out using DEGs for enrichment analysis.

Values of variables with a normal distribution are expressed as mean ± standard deviation (SD) and were analyzed using a two-tailed unpaired *t*-test. A *p*-value of <0.05 was considered statistically significant. GraphPad Prism 10 (San Diego, California, United States) was used for graphing and statistical analysis.

### 2.2 Single-cell transcriptomic analysis

#### 2.2.1 Processing of raw data, cell filtering, and classification of single-cell subpopulations

The number of reads obtained from the 10 × Genomics platform for psoriasis (PS) and DBP samples were 345,355,552 and 334,982,898, respectively. The Q30 scores of RNA reads were both >94.2%, indicating high read quality for subsequent analysis. In the PS and DBP groups, 9,693 and 11,981 cells were identified, respectively, which was sufficient for further cell classification. After filtering, 7,572 PS cells and 10,241 DBP cells remained for further study. Overall, 97% of reads were associated with GRCh38/GRCm38, indicating that the data were not contaminated and thus were reliable. While biological replicates from several patients were not feasible in this case study, rigorous technical controls throughout the process was performed. The high cell viability (>85%) and sequencing quality scores (Q30 > 94.2%) support the reliability of the single-cell data. All computational analyses followed established pipelines with predetermined thresholds so as to ensure reproducibility.

#### 2.2.2 Cell grouping

**Figure 2:**
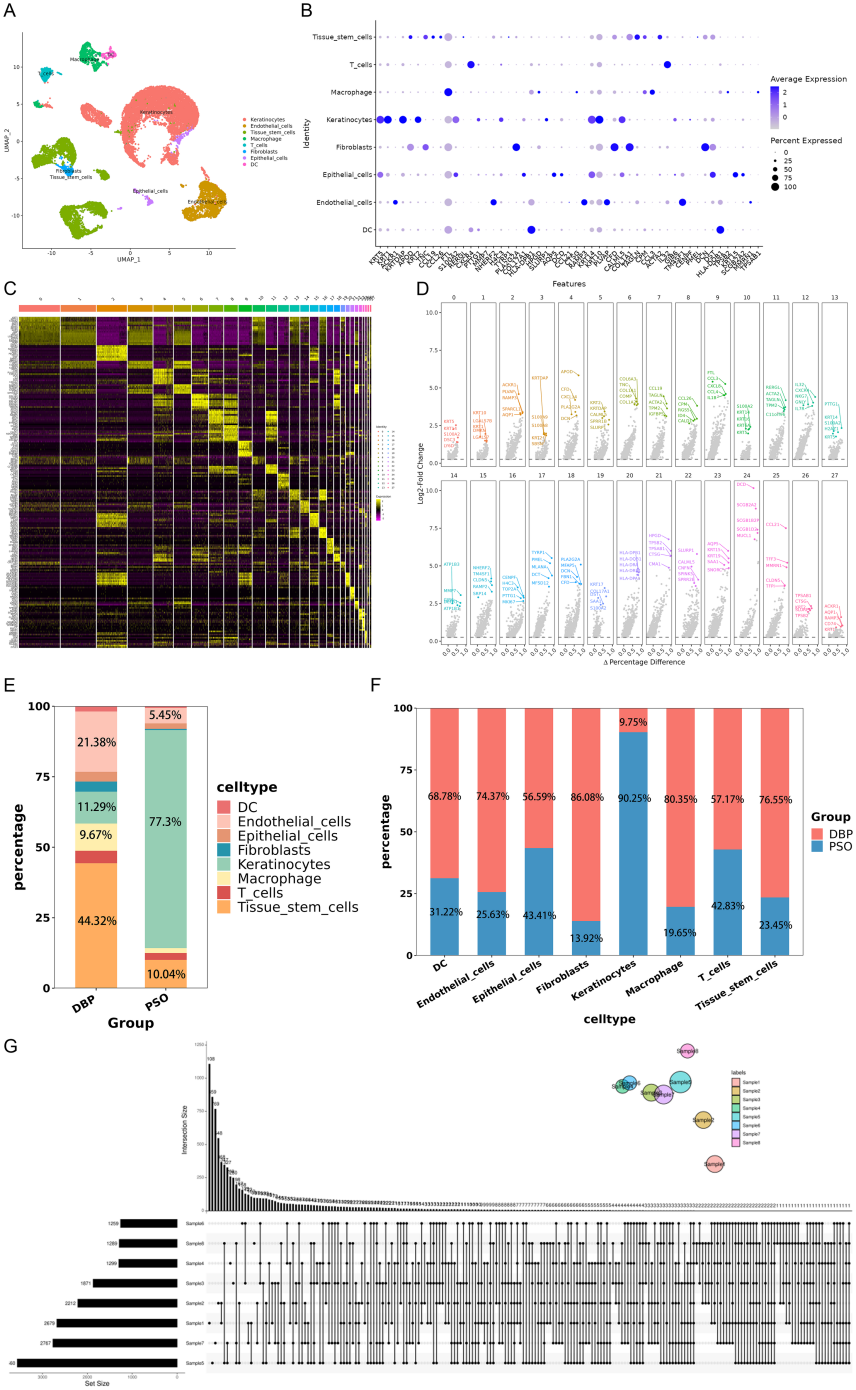
Cell clustering and marker gene identification. **(A)** t-SNE clustering of snRNA-Seq data. Cell types were annotated based on known marker gene expression and visualized by merging t-SNE plots of identical cells. **(B)** t-SNE plot showing the expression of classical marker genes. Each point represents a cell. Darker color intensity indicates higher expression levels of the marker gene in that cell. **(C)** Heatmap of marker genes in each cluster. **(D)** Top five differentially expressed genes in each cluster. **(E)** Bar chart depicting the number of cells. **(F)** Bar chart depicting the detailed ratio of cells in samples. **(G)** The upset plot of DEGs across all clusters.

After strict quality control, the remaining cells were annotated as different cell types based on known marker genes (Figure 2A). Cells were then classified into 27 clusters and grouped into 8 categories: T cells, fibroblasts, keratinocytes, macrophages, endothelial cells, epithelial cells, dendritic cells, and tissue stem cells were recognized as marker genes (Figure 2B). Furthermore, in each cluster, genes with high specificity and expression (log_2_*FC* > 0.25), especially in at least 25% of cells, were identified as marker genes (Figure 2C). The top five genes were identified in each cluster (Figure 2D).

#### 2.2.3 Differences in cluster distribution between the two groups

By comparing the distribution of cell types between the two groups, the cell types were identified with differential distribution specific to each group. Tissue stem cells (44.32%) were the most predominant in DBP, followed by endothelial cells (21.38%). In the psoriasis (PSO) group, keratinocytes (77.3%) were the most abundant, followed by epithelial cells (10.04%) and endothelial cells (5.45%). The composition of cell types differed between the DBP and PSO groups at the single-cell level (Figure 2E). More specifically, dendritic cells (68.78% vs. 31.22%), endothelial cells (74.37% vs. 25.63%), epithelial cells (56.59% vs. 43.41%), fibroblasts (86.08% vs. 13.92%), macrophages (80.35% vs. 19.65%), T cells (57.17% vs. 42.83%), and tissue stem cells (76.55% vs. 23.45%) were more prevalent in DBP than in PSO, whereas keratinocytes (90.25% vs. 9.75%) were more prevalent in PSO than in DBP (Figure 2F).

#### 2.2.4 Identification of DEGs in cells

The differentially expressed genes (DEGs) were analyzed in the eight cell types, with 2,680 identified in keratinocytes, 2,213 in epithelial cells, 1,872 in macrophages, 1,300 in T cells, 1,260 in dendritic cells, 2,768 in endothelial cells, 1,290 in fibroblasts, and 3,561 in tissue stem cells. Among all DEGs, three genes were commonly expressed in all cell types, namely MT-ND2, MALAT1, and NDFIP1 (Figure 2G).

#### 2.2.5 Identification and functional analysis of DEGs in keratinocytes

Among the 684 DEGs identified in keratinocytes, 435 were upregulated and 249 were downregulated (Supplementary Figure S2A). The most upregulated DEGs were KRT6B, KRT6C, MMP7, KRT17, KRT6A, FGFBP1, SAT1, S100A2, KRT16, and ANXA1, while the most downregulated DEGs were KRT2, KRT1, KRT10, KRTDAP, SLURP1, APOE, DMKN, LGALS7B, DSC1, and CALML3.

The top 10 enriched GO terms for keratinocyte DEGs included protein binding, extracellular exosome, cytoplasm, cytosol, nucleus, ribonucleoprotein complex, RNA binding, cytoplasmic translation, cell membrane, and ribosome (Supplementary Figure S2B).

The top 10 enriched KEGG pathways for keratinocyte DEGs were the ribosome pathway in human genetic information processing, coronavirus disease, Parkinson’s disease, amyotrophic lateral sclerosis (ALS), prion disease, neurodegeneration, Alzheimer’s disease, metabolic pathways, Huntington’s disease (HD), and oxidative phosphorylation (Supplementary Figure S2C).

The transcriptomic profile of keratinocytes in BP lesions exhibited a remarkable shift from differentiation to activation, which was characterized by the concerted upregulation of stress-associated keratins KRT6B and KRT6C alongside the downregulation of differentiation markers KRT1 and KRT10. This molecular pattern revealed a state of keratinocyte injury and repair, further improved by the overexpression of MMP7, a protease known to degrade key basement membrane components. These changes collectively suggested a mechanism in which the keratinocytes actively contribute to blister formation through impaired epidermal integrity and targeted enzymatic disruption of the dermal–epidermal junction.

#### 2.2.6 Identification and functional analysis of DEGs in epithelial cells

The analysis identified 1,028 DEGs in epithelial cells, of which 673 were upregulated and 355 were downregulated (Supplementary Figure S3A). The most upregulated genes included KRT6A, IFI6, S100A10, S100A2, IFI27, FGFBP1, ISG15, RAP2B, KRT16, and TMSB4X, while the most downregulated genes included PIP, DCD, MUCL1, SCGB1B2P, KRT1, KRT10, SCGB1D2, SCGB2A2, KRTDAP, and KRT2.

The top 10 enriched GO terms for epithelial cell DEGs were protein binding, extracellular exosome, cytoplasm, cytosol, nucleus, RNA binding, membrane, adhesion, extracellular region, and extracellular space (Supplementary Figure S3B).

The top 10 enriched KEGG pathways for epithelial cell DEGs were coronavirus disease, ribosome, Salmonella infection, prion disease, metabolic pathways, amyotrophic lateral sclerosis (ALS), Parkinson’s disease, pathways in neurodegenerative diseases, Huntington’s disease, and Alzheimer’s disease (Supplementary Figure S3C).

The epithelial cells in BP lesions demonstrated a pronounced interferon response signature, highlighting the strong upregulation of IFI6, IFI27, and ISG15. This was further accompanied by a stress and activation profile evidenced by increased levels of KRT6A and S100A family proteins. Concurrently, the loss of structural and secretory markers such as PIP and DCD was observed. This transcriptomic shift suggests that epithelial cells contribute to BP pathogenesis by increasing local inflammation through interferon signaling, while simultaneously undergoing a functional alteration that compromises their specialized roles in skin homeostasis.

#### 2.2.7 Identification and functional analysis of DEGs in macrophages

The analysis identified 1,111 DEGs in macrophages, of which 567 were upregulated and 544 were downregulated (Supplementary Figure S4A). The most upregulated genes were FABP4, ISG15, CTSL, SPP1, FABP5, IFI6, PHLDA1, EREG, C15orf48, and CCL4. The most downregulated genes were KRT1, DMKN, KRT10, KRTDAP, KRT14, C1QA, PERP, CCL17, KRT5, and C1QB.

The top 10 enriched GO terms for macrophage DEGs were extracellular exosome, protein binding, cytoplasm, cytoplasmic translation, cytosol, membrane, cytoplasmic ribosome, ribosomal structure, ribosome, and protein translation (Supplementary Figure S4B).

The top 10 enriched KEGG pathways for macrophage DEGs were coronavirus disease, ribosome, rheumatoid arthritis, phagosome, Salmonella infection, tuberculosis, influenza A, EB virus infection, antigen processing and presentation, and Shigella disease (Supplementary Figure S4C).

Macrophages in BP lesions displayed a clear pro-inflammatory polarization, evidenced by the upregulation of key mediators such as SPP1, which is linked to M1 macrophage activation, and the chemokine CCL4, which is involved in lymphocyte recruitment. The concurrent downregulation of complement components C1QA and C1QB suggested a potential shift in their phagocytic and immunoregulatory functions. This transcriptional reprogramming demonstrated macrophages as active drivers of inflammation in BP, contributing to the overall immune dysregulation through cytokine signaling and altered innate immune responses.

#### 2.2.8 Identification and functional analysis of DEGs in T cells

The analysis identified 477 DEGs in T cells, including 284 upregulated and 193 downregulated genes (Supplementary Figure S5A). The most upregulated DEGs, based on fold change, were KLRD1, MTATP6P1, IFI6, CEBPB, RGS1, RGCC, CD247, GZMB, BST2, and TYROBP. The most downregulated DEGs were KRT10, KRT1, DMKN, KRTDAP, LY6D, SFN, S100A8, KRT2, LGALS7B, and DSP.

Functional enrichment analysis revealed that the top 10 enriched GO terms for T-cell DEGs were protein binding, cytoplasmic translation, cytoplasmic ribosomes, ribonucleoprotein complexes, cytoplasm, ribosomal structures, ribosomes, RNA binding, extracellular exosomes, and cytoplasmic large ribosomal subunits (Supplementary Figure S5B).

KEGG pathway enrichment analysis showed that the top 10 enriched pathways for T cell DEGs were ribosome, coronavirus disease, Parkinson’s disease, oxidative phosphorylation, thermogenesis, prion disease, Huntington’s disease, pathways in neurodegenerative diseases, Alzheimer’s disease, and amyotrophic lateral sclerosis (Supplementary Figure S5C).

T cells in BP lesions exhibited a cytotoxic and interferon-activated phenotype, supported by the marked upregulation of granzyme B (GZMB), a key effector molecule of cytotoxic activity, alongside CD247, a central component of the T-cell receptor complex. The upgradation of IFI6 further indicated strong interferon signaling within these cells. This transcriptional profile suggests that T cells may contribute directly to tissue damage in BP through cytotoxic mechanisms, while also participating in the broader inflammatory cascade driven by interferon activation.

#### 2.2.9 Identification and functional analysis of DEGs in dendritic cells

The analysis identified 653 DEGs in dendritic cells, including 305 upregulated and 348 downregulated genes (Supplementary Figure S6A). The most upregulated DEGs were GZMB, CCL22, CCR7, ISG20, CST7, ISG15, IL7R, TXN, NR4A3, and IDO1. The most downregulated DEGs were KRT1, KRT10, KRT14, DMKN, KRT5, KRTDAP, LY6D, DSP, KRT2, and SFN.

The top 10 enriched GO terms for dendritic cell DEGs were protein binding, cytoplasmic translation, membrane, extracellular exosomes, cytoplasmic ribosomes, ribosomal structures, ribonucleoprotein complexes, ribosomes, cytoplasmic large ribosomal subunits, and cytoplasm (Supplementary Figure S6B).

KEGG pathway enrichment analysis indicated that the top 10 enriched pathways for dendritic cell DEGs were coronavirus disease, ribosome, prion disease, Parkinson’s disease, cancer-related pathways, pathways in neurodegenerative diseases, oxidative phosphorylation, Kaposi’s sarcoma-associated herpesvirus infection, amyotrophic lateral sclerosis, and Alzheimer’s disease (Supplementary Figure S6C).

Dendritic cells in BP lesions demonstrated features of immune activation and regulation. The upregulation of CCL22 and its receptor CCR7 pointed to an active role in T-cell recruitment and lymphoid homing. The presence of GZMB suggested a non-canonical cytotoxic potential, while IDO1 expression indicated immunomodulatory activity. These results demonstrated dendritic cells as pivotal coordinators in BP pathogenesis, bridging innate and adaptive immune responses through chemokine signaling and direct immune regulation.

#### 2.2.10 Identification and functional analysis of DEGs in endothelial cells

We identified 617 DEGs in endothelial cells, including 305 upregulated genes, such as SOCS3, C2CD4B, CCL2, IER3, IFI6, MIR23AHG, HES1, ZFP36, ADAMTS1, and CRIP1, and 348 downregulated DEGs, including KRT1, CCL21, DMKN, KRT10, KRTDAP, KRT14, CCL23, CLU, S100A9, and S100A8 (Supplementary Figure S7A). Following the identification of DEG, GO and KEGG pathway enrichment analyses were performed. The top 10 enriched GO terms for endothelial cell DEGs included protein binding, extracellular exosome, cytoplasm, cytosol, cytoplasmic translation, cytoplasmic ribosome, nucleus, ribonucleoprotein complex, focal adhesion plaques, and ribosomal structures (Supplementary Figure S7B). The top 10 enriched KEGG pathways included coronavirus disease 2019 (COVID-19), ribosome, Parkinson’s disease, human T cell leukemia virus 1 infection, influenza A, Epstein–Barr virus (EBV) infection, Shigellosis, Alzheimer’s disease, Salmonella infection, and Th17 cell differentiation (Supplementary Figure S7C).

Endothelial cells in BP lesions exhibited a distinct pro-inflammatory activation state. This was categorized by the upregulated expression of the key chemokine CCL2, a potent monocyte recruiter, and SOCS3, a regulator of inflammatory signaling. The downregulation of CCL21, a chemokine involved in lymphocyte homing, suggested an altered leukocyte trafficking pattern. This transcriptional profile implied that endothelial cells actively contributed to the inflammatory milieu in BP by modulating the recruitment and migration of specific immune cell subsets into the lesions.

#### 2.2.11 Identification and functional analysis of DEGs in fibroblasts

The analysis identified 1,885 DEGs in fibroblasts, including 859 upregulated genes such as PTX3, PLA2G2A, IFI6, RARRES1, KLF2, PRSS23, RGCC, JUN, HSPA1A, and PCOLCE2, and 1,026 downregulated DEGs including KRT14, KRT1, KRT5, DMKN, KRT16, LY6D, KRT10, SFN, S100A9, and S100A8 (Supplementary Figure S8A).

The subsequent GO and KEGG pathway enrichment analyses revealed that the top 10 enriched GO terms for fibroblast DEGs were protein binding, extracellular exosome, cytoplasm, cytosol, membrane, nucleus, RNA binding, nucleoplasm, focal adhesion plaques, and extracellular region (Supplementary Figure S8B).

The top 10 enriched KEGG pathways were ribosome, metabolic pathways, pathways in neurodegenerative diseases, coronavirus disease 2019 (COVID-19), Parkinson’s disease, amyotrophic lateral sclerosis, Alzheimer’s disease, Huntington’s disease, endocytosis, and prion disease (Supplementary Figure S8C).

Fibroblasts in BP lesions adopted a pro-fibrotic and inflammatory phenotype. This was strongly specified by the upregulation of PTX3, a mediator of complement activation and matrix remodeling, and JUN, a key transcription factor in stress responses. HSPA1A expression further suggested cellular stress. This transcriptional shift implied that fibroblasts actively contributed to BP pathogenesis by driving extracellular matrix reorganization and modifying local inflammatory circuits.

#### 2.2.12 Identification and functional analysis of DEGs in tissue stem cells

The analysis identified 683 DEGs in tissue stem cells, including 403 upregulated and 280 downregulated (Supplementary Figure S9A). The top 10 most upregulated DEGs were IFI6, ISG15, CCL8, PLA2G2A, TIMP1, IFITM1, TNC, SERPINE2, ID4, and PTX3, and the top 10 most downregulated DEGs were KRT1, DMKN, KRT10, KRTDAP, LY6D, S100A8, S100A9, KRT14, SFN, and DSP.

The top 10 enriched GO terms for tissue stem cell DEGs were extracellular exosomes, protein binding, extracellular region, extracellular space, cytoplasm, collagen-containing extracellular matrix, focal adhesion plaques, cytoplasmic translation, cytosol, and cytoplasmic ribosome (Supplementary Figure S9B).

The top 10 enriched KEGG pathways for tissue stem cell DEGs were COVID-19, ribosome, Alzheimer’s disease, Parkinson’s disease, cancer pathway, PI3K-Akt signaling pathway, pathway in neurodegenerative disease, Salmonella infection, and protein processing in the endoplasmic reticulum (Supplementary Figure S9C).

Tissue stem cells in BP lesions exhibited a pronounced inflammatory activation and matrix remodeling signature. This state was distinct from the upregulation of key mediators, including the interferon response gene IFI6, the chemokine CCL8, and the matrix regulator PTX3. The concurrent induction of TIMP1 and TNC further showed active extracellular matrix reorganization. This transcriptional profile proposed that tissue stem cells contributed to BP pathogenesis by modifying immune responses through cytokine signaling and disrupting tissue integrity through aberrant matrix remodeling.

## 3 Discussion

BP is an autoimmune subepidermal disorder characterized by pruritus, followed by edematous plaques and eventual blister formation on the skin and mucous membranes. BP develops when autoantibodies target two hemidesmosomal proteins, BP230 and BP180, leading to complement cascade activation, inflammatory cell migration, and subepidermal blister formation (8).

This study described a case of BP occurring immediately after the initiation of guselkumab in an elderly patient with psoriasis. While advanced age is a known risk factor for BP, the temporal sequence of symptom onset within 2 weeks of the first dose, the absence of other new or changed concomitant medications, and the rapid resolution upon drug withdrawal collectively provided strong clinical evidence for a drug-induced etiology. This causality was further supported by a Naranjo algorithm score of 7. Therefore, this case represented a probable instance of BP induced by guselkumab. Single-cell RNA sequencing revealed specific transcriptomic differences among cell types between psoriatic and BP lesions. Although anti-IL-23 antibodies have demonstrated significant therapeutic efficacy in psoriasis, our study suggests that these antibodies have the potential to trigger BP via immune dysregulation. BP, an autoimmune blistering disorder characterized by epidermal–dermal junction separation, is typically associated with anti-BP180/BP230 antibodies. Seronegativity in this case, as determined by ELISA systems targeting the NC16A domain of BP180 and BP230, represents a recognized phenomenon in a subset of BP cases, particularly in drug-induced variants. It is well-documented that certain BP cases, including those induced by medications such as gliptins, may exhibit negative results on standard ELISA due to autoantibodies targeting epitopes outside the immunodominant NC16A domain (9). Therefore, the absence of circulating antibodies through standard ELISA in the patient does not preclude the diagnosis of BP. Instead, it strengthens the classification as a drug-induced variant, where the clinical and histopathological profile, coupled with this specific serological characteristic, aligns with the established patterns of DBP. We acknowledge that direct immunofluorescence, the diagnostic gold standard for BP, was not carried out in this study. Nevertheless, the diagnosis of DBP remains highly plausible based on the confluence of key evidence: the classic clinical presentation of generalized bullae, the definitive histopathological demonstration of a subepidermal split with eosinophilic infiltration, the compelling temporal link to guselkumab initiation, and the rapid, complete response to drug withdrawal and corticosteroid therapy. This diagnostic approach, based on a combination of clinical and histopathological criteria in the absence of serological or immunofluorescence confirmation, is well-established in the literature for similar cases of DBP. Guselkumab, a monoclonal antibody targeting the IL-23p19 subunit, exhibits therapeutic effects by inhibiting Th17 cell differentiation. Its broad immunomodulatory effects, including modulation of Th17 and related immune pathways, may disrupt self-tolerance, precipitating BP. The eruption’s temporal onset 2 weeks after treatment initiation, together with its prompt reversal upon corticosteroid therapy, is consistent with DBP (10).

The occurrence of BP following guselkumab therapy is not unprecedented and should be considered within the broader context of IL-23 inhibition. Several case reports have documented similar paradoxical reactions. A case report consistent with our case described guselkumab-associated BP in a psoriasis patient, also presenting with negative BP180/BP230 antibodies, highlighting the seronegative phenotype as a potential feature (3). Furthermore, this phenomenon appears to extend beyond guselkumab. Instances of BP have been associated with risankizumab, another anti-IL-23p19 inhibitor (3), and are more frequently associated with ustekinumab, which targets the IL-12/IL-23 p40 subunit (11). This pattern across multiple biologics that suppress the IL-23 pathway suggests a potential class effect. The underlying mechanism may involve a significant immunological shift. By potently inhibiting the IL-23/Th17 axis, these drugs may inadvertently facilitate a Th2-polarized immune response, which is central to the pathogenesis of BP (12). This shift could potentially lower the threshold for B-cell activation and the production of autoantibodies, including those targeting non-NC16A epitopes, thereby explaining the seronegative status in some of these drug-induced cases.

Prior to detailing the specific transcriptomic findings, it is essential to contextualize the nature of this analysis. The scRNA-seq data presented hereafter provided a high-resolution, yet singular, perspective from one individual. The patient’s comorbidities of type 2 diabetes and hyperlipidemia, while stable, introduce potential confounding variables that must be considered when taking the differential gene expression patterns. Consequently, the ensuing analysis serves to generate mechanistic hypotheses centered on this specific clinical event, and these hypotheses require substantiation in broader patient cohorts.

Significantly, scRNA-seq analysis comparing cellular composition and transcriptomic profiles between psoriatic and BP lesions in the same patient revealed significant enrichment of tissue stem cells (44.3%) and endothelial cells (21.4%) in BP lesions, whereas psoriatic lesions were dominated by keratinocytes (77.3%). This finding reflects their distinct pathogenic mechanisms: psoriasis centers on keratinocyte hyperproliferation, while BP involves aberrant tissue repair and immune cell infiltration.

In BP lesions, keratinocytes showed abnormal activation with upregulated KRT6B, KRT6C, and MMP7 and with downregulated differentiation-related genes (KRT1 and KRT10). KRT6 regulates collective keratinocyte migration via cell–cell and cell-matrix adhesion modulation; its overexpression is observed in cutaneous injury and inflammation (13). MMP7 (matrilysin), a member of the matrix metalloproteinase family and a zinc-dependent endopeptidase, degrades extracellular matrix (ECM) components such as collagen and laminin (14). Thus, KRT6 may influence BP pathogenesis through adhesion modulation, while MMP7 promotes blister formation by degrading the basement membrane.

Distinct patterns of immune cell regulation in BP lesions comprises upregulated pro-inflammatory genes (SPP1 and GZMB) in macrophages and T cells. SPP1 (osteopontin) promotes M1 macrophage polarization and cytokine release, while GZMB (granzyme B) facilitates cytotoxic T cell-induced tissue damage (15). These cells exhibited elevated CCL22 and CCR7 expression, potentially driving T-cell recruitment. The upregulation of IFI6 across multiple cell types was a prominent feature, potentially implicating the activation of the interferon pathway as a contributor to BP pathogenesis following guselkumab exposure.

Tissue stem cells predominated in BP lesions (76.6% vs. 23.5%). Their differentially expressed genes (DEGs), including TIMP1 and PTX3 (pentraxin 3), highlight stem cell dysregulation and extracellular matrix remodeling. The observed elevation of PTX3, a molecule capable of activating complement and promoting eosinophil infiltration (16), offers a plausible correlate for the eosinophilic infiltration characteristic of BP. Enrichment of ECM-related pathways, such as PI3K-Akt signaling, suggests that the microenvironmental imbalance exacerbates epidermal-dermal separation via mechanotransduction.

Unlike other biologics that target downstream pathways, guselkumab specifically inhibits IL-23 to target upstream Th17 differentiation. The inhibition of IL-23 may initiate Th1/Th2 responses and dysregulate autoreactive B cells, thereby producing autoantibodies against cutaneous structures (17). Although classic BP antibodies were not detected, the scRNA-seq analysis revealed elevated B-cell activation markers (CD79A), suggesting the involvement of humoral immunity.

**Figure 3:**
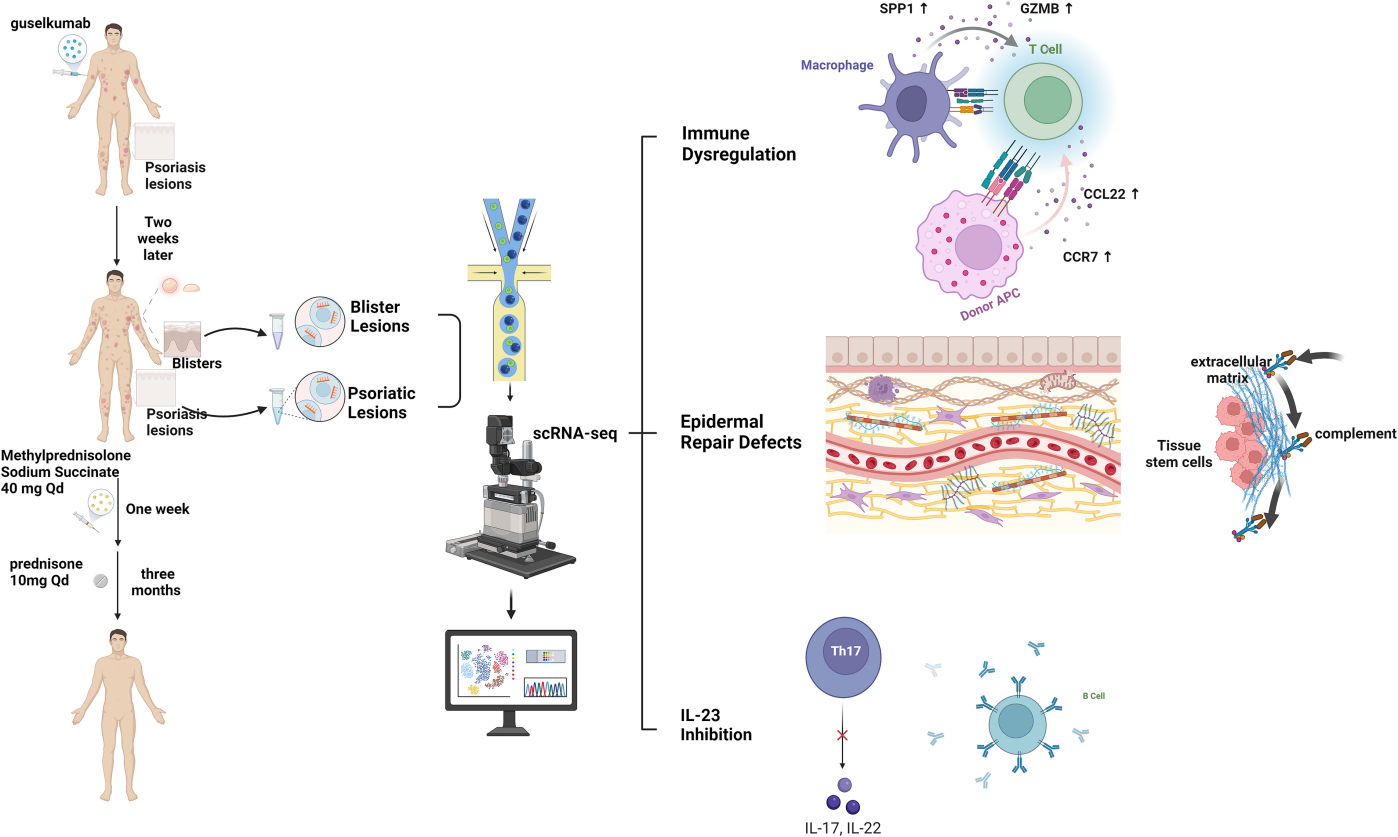
Proposed molecular and cellular mechanism of guselkumab-induced bullous pemphigoid. Guselkumab-mediated IL-23 inhibition triggers a dual pathogenic pathway: (1) Immune dysregulation: skewing toward a Th2-type response and broad activation of interferon signaling (e.g., IFI6), promoting B-cell activation and autoantibody production. (2) Defective epidermal repair: Keratinocytes undergo stress and aberrant activation (upregulation of KRT6B/C, MMP7), while tissue stem cells (TSCs) contribute to extracellular matrix (ECM) remodeling and complement activation via PTX3. These parallel processes synergize to cause eosinophil recruitment, basement membrane degradation, and subepidermal blistering. Created with BioRender.com.

Collectively, these findings demonstrated that the inhibition of IL-23 by guselkumab disrupts immune homeostasis, potentially facilitating a Th2-skewed response and an interferon signature across multiple cell types. Concurrently, keratinocytes exhibit a stress response characterized by KRT6 and MMP7 upregulation, while tissue stem cells show increased PTX3 expression. These alterations collectively contribute to basement membrane degradation, complement activation, eosinophilic infiltration, and subepidermal blister formation. This visual summary encapsulates the proposed “dual-hit” hypothesis of immune dysregulation and impaired epidermal repair constituting the core of this study (Figure 3).

Based on these immune dysregulation findings, scRNA-seq analysis identified MT-ND2, MALAT1, and NDFIP1 as the only DEGs co-expressed across all cell types. MT-ND2, a mitochondrial respiratory chain component, may enhance epidermal–dermal separation through the accumulation of reactive oxygen species (ROS) (18). MALAT1, a long non-coding RNA, may contribute to immune dysregulation by modulating inflammatory gene expression (19). NDFIP1, a ubiquitin ligase adaptor, likely participates in extracellular matrix (ECM) remodeling and the regulation of inflammatory cell infiltration (20). Functional validation and multi-omics integration are required to elucidate the molecular mechanisms underlining the roles of these genes.

The findings should be interpreted under the context of their exploratory nature. The primary constraint of this study is its reliance on a single case, which restricts the generalizability of the transcriptomic signatures described. Although internally consistent and biologically informative, these findings are specific to particular patient’s unique genetic background and clinical context, including comorbidities. Future research should therefore prioritize the recruitment of larger cohorts of patients experiencing similar paradoxical reactions. Such studies will further enable the effective distinction of core pathogenic mechanisms from patient-specific variations. Moreover, the functional significance of key candidate genes, such as IFI6 and PTX3, remains to be experimentally validated using relevant model systems. Advanced techniques, such as spatial transcriptomics, could provide deeper insights into the cellular interactions driving this adverse event.

## 4 Conclusion

This study offers insights into the molecular mechanisms underlying guselkumab-induced BP, highlighting immune dysregulation and impaired epidermal repair. Larger cohorts and multi-modal analyses are required for the validation of these findings.

## Data Availability

The original contributions presented in the study are included in the article/Supplementary material, further inquiries can be directed to the corresponding author.

## Supplementary material

SUPPLEMENTARY FIGURE S1 Schematic representation of lesion sampling for single-cell RNA sequencing. **(A)** Areas marked indicate the specific psoriatic lesions from which tissue samples were obtained for comparative transcriptomic analysis. **(B)** Areas marked indicate the specific bullous pemphigoid lesions from which tissue samples were obtained for comparative transcriptomic analysis.

SUPPLEMENTARY FIGURE S2 Identification differentially expressed genes (DEGs) and functional analysis of DEGs in keratinocytes. **(A)** The heatmap of DEGs in keratinocytes. **(B)** The gene ontology (GO) analysis of DEGs in keratinocytes. **(C)** The Kyoto encyclopedia of genes and genomes (KEGG) enrichment of DEGs in keratinocytes.

SUPPLEMENTARY FIGURE S3 Identification differentially expressed genes (DEGs) and functional analysis of DEGs in epithelial cells. **(A)** The heatmap of DEGs in epithelial cells. **(B)** The gene ontology (GO) analysis of DEGs in epithelial cells. **(C)** The Kyoto encyclopedia of genes and genomes (KEGG) enrichment of DEGs in epithelial cells.

SUPPLEMENTARY FIGURE S4 Identification differentially expressed genes (DEGs) and functional analysis of DEGs in macrophages. **(A)** The heatmap of DEGs in macrophages. **(B)** The gene ontology (GO) analysis of DEGs in macrophages. **(C)** The Kyoto encyclopedia of genes and genomes (KEGG) enrichment of DEGs in macrophages.

SUPPLEMENTARY FIGURE S5 Identification differentially expressed genes (DEGs) and functional analysis of DEGs in T cells. **(A)** The heatmap of DEGs in T cells. **(B)** The gene ontology (GO) analysis of DEGs in T cells. **(C)** The Kyoto encyclopedia of genes and genomes (KEGG) enrichment of DEGs in T cells.

SUPPLEMENTARY FIGURE S6 Identification differentially expressed genes (DEGs) and functional analysis of DEGs in dendritic cells. **(A)** The heatmap of DEGs in dendritic cells. **(B)** The gene ontology (GO) analysis of DEGs in dendritic cells. **(C)** The Kyoto encyclopedia of genes and genomes (KEGG) enrichment of DEGs in dendritic cells.

SUPPLEMENTARY FIGURE S7 Identification differentially expressed genes (DEGs) and functional analysis of DEGs in endothelial cells. **(A)** The heatmap of DEGs in endothelial cells. **(B)** The gene ontology (GO) analysis of DEGs in endothelial cells. **(C)** The Kyoto encyclopedia of genes and genomes (KEGG) enrichment of DEGs in endothelial cells.

SUPPLEMENTARY FIGURE S8 Identification differentially expressed genes (DEGs) and functional analysis of DEGs in fibroblasts. **(A)** The heatmap of DEGs in fibroblasts. **(B)** The gene ontology (GO) analysis of DEGs in fibroblasts. **(C)** The Kyoto encyclopedia of genes and genomes (KEGG) enrichment of DEGs in fibroblasts.

SUPPLEMENTARY FIGURE S9 Identification differentially expressed genes (DEGs) and functional analysis of DEGs in tissue stem cells. **(A)** The heatmap of DEGs in tissue stem cells. **(B)** The gene ontology (GO) analysis of DEGs in tissue stem cells. **(C)** The Kyoto encyclopedia of genes and genomes (KEGG) enrichment of DEGs in tissue stem cells.
