# Supplementary figures and images for: Bullous pemphigoid induced by anti-IL-23 monoclonal antibody in a psoriatic patient: a case report
Source: Front Med (Lausanne). 2025 Dec 18;12:1709423. doi: 10.3389/fmed.2025.1709423 (PMC12756085; doi:10.3389/fmed.2025.1709423)

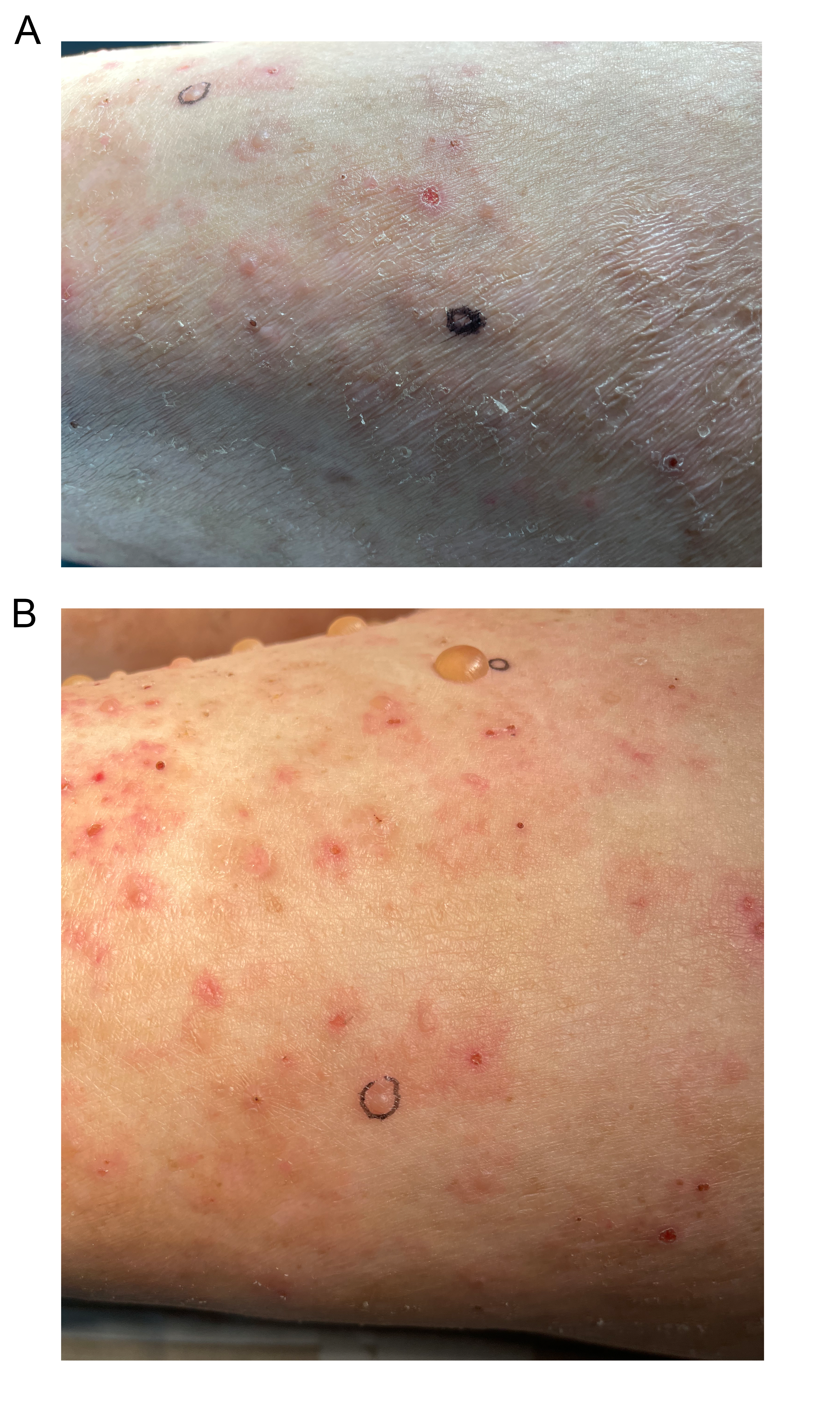

Supplement: SUPPLEMENTARY FIGURE S1 — Schematic representation of lesion sampling for single-cell RNA sequencing. (A) Areas marked indicate the specific psoriatic lesions from which tissue samples were obtained for comparative transcriptomic analysis. (B) Areas marked indicate the specific bullous pemphigoid lesions from which tissue samples were obtained for comparative transcriptomic analysis. [file Image_1.TIF]

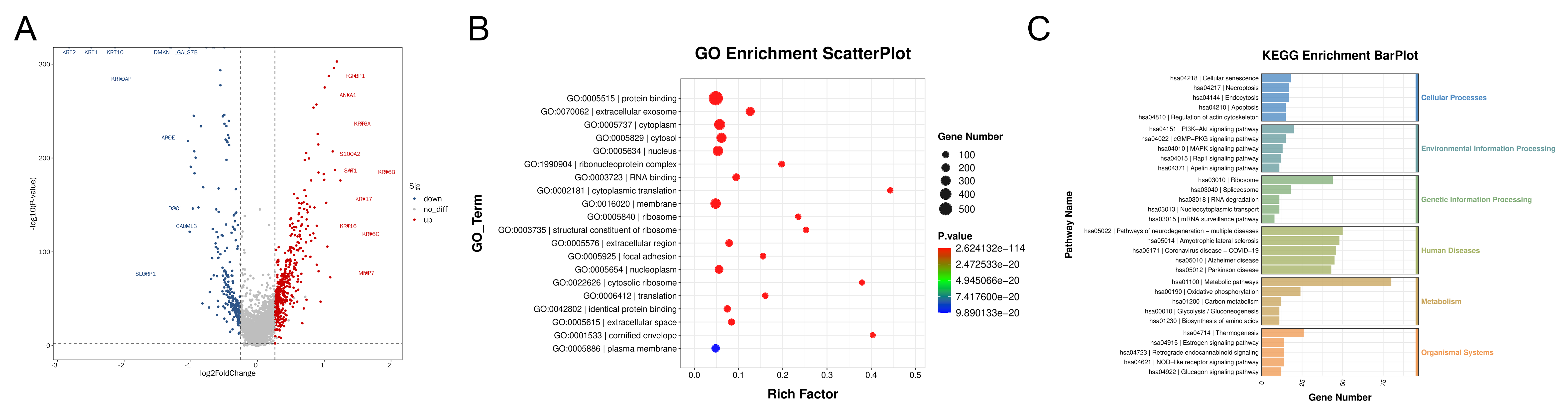

Supplement: SUPPLEMENTARY FIGURE S2 — Identification differentially expressed genes (DEGs) and functional analysis of DEGs in keratinocytes. (A) The heatmap of DEGs in keratinocytes. (B) The gene ontology (GO) analysis of DEGs in keratinocytes. (C) The Kyoto encyclopedia of genes and genomes (KEGG) enrichment of DEGs in keratinocytes. [file Image_2.TIF]

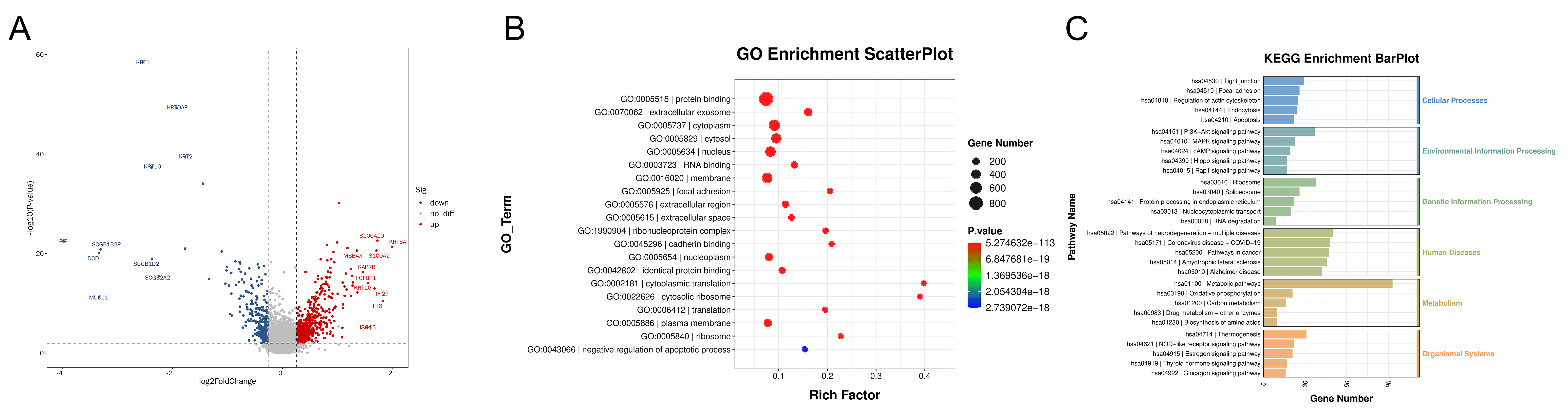

Supplement: SUPPLEMENTARY FIGURE S3 — Identification differentially expressed genes (DEGs) and functional analysis of DEGs in epithelial cells. (A) The heatmap of DEGs in epithelial cells. (B) The gene ontology (GO) analysis of DEGs in epithelial cells. (C) The Kyoto encyclopedia of genes and genomes (KEGG) enrichment of DEGs in epithelial cells. [file Image_3.TIF]

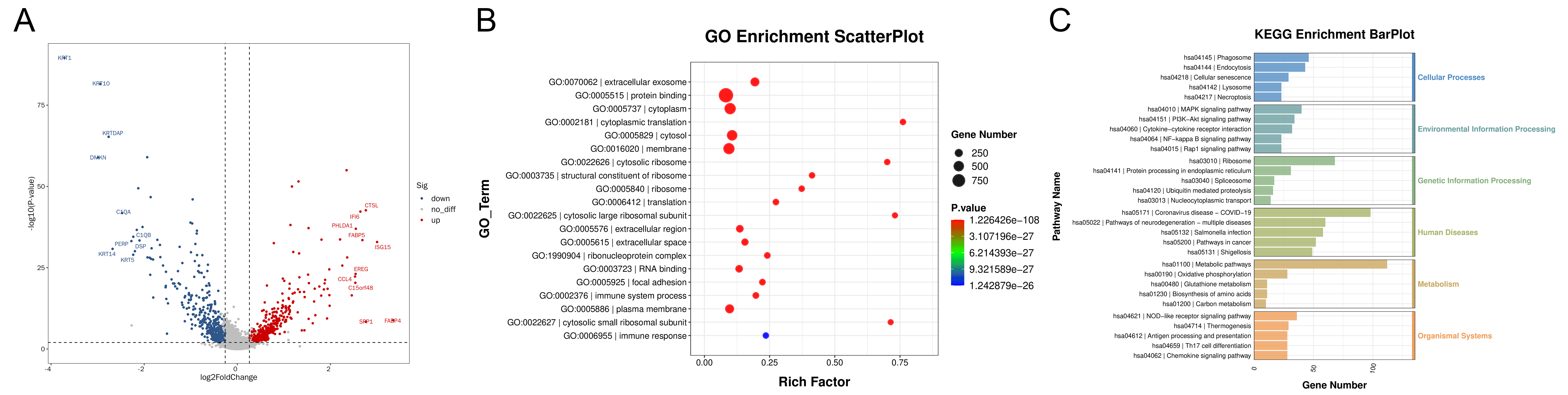

Supplement: SUPPLEMENTARY FIGURE S4 — Identification differentially expressed genes (DEGs) and functional analysis of DEGs in macrophages. (A) The heatmap of DEGs in macrophages. (B) The gene ontology (GO) analysis of DEGs in macrophages. (C) The Kyoto encyclopedia of genes and genomes (KEGG) enrichment of DEGs in macrophages. [file Image_4.TIF]

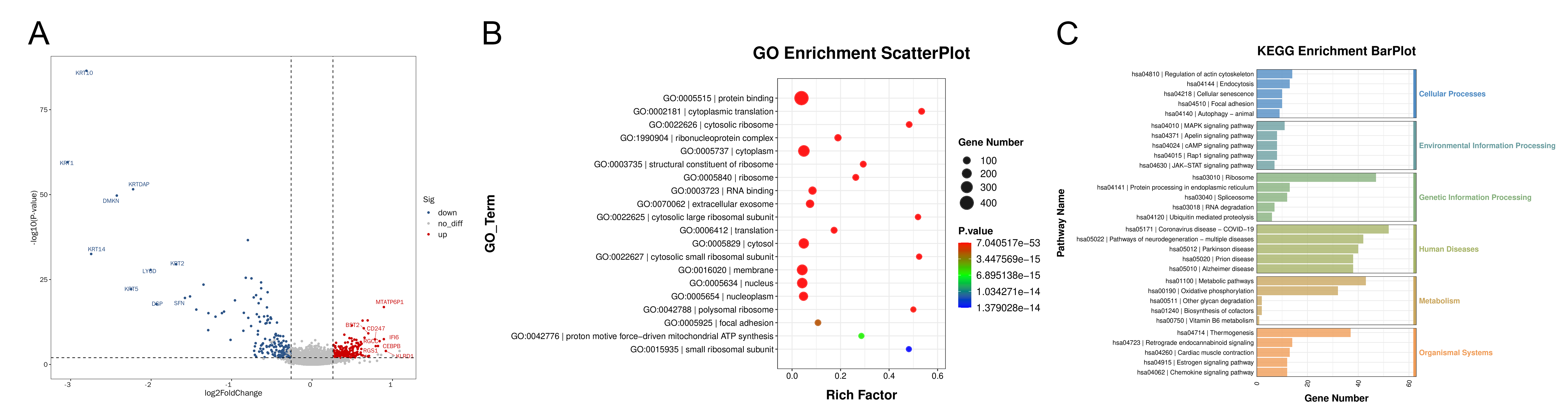

Supplement: SUPPLEMENTARY FIGURE S5 — Identification differentially expressed genes (DEGs) and functional analysis of DEGs in T cells. (A) The heatmap of DEGs in T cells. (B) The gene ontology (GO) analysis of DEGs in T cells. (C) The Kyoto encyclopedia of genes and genomes (KEGG) enrichment of DEGs in T cells. [file Image_5.TIF]

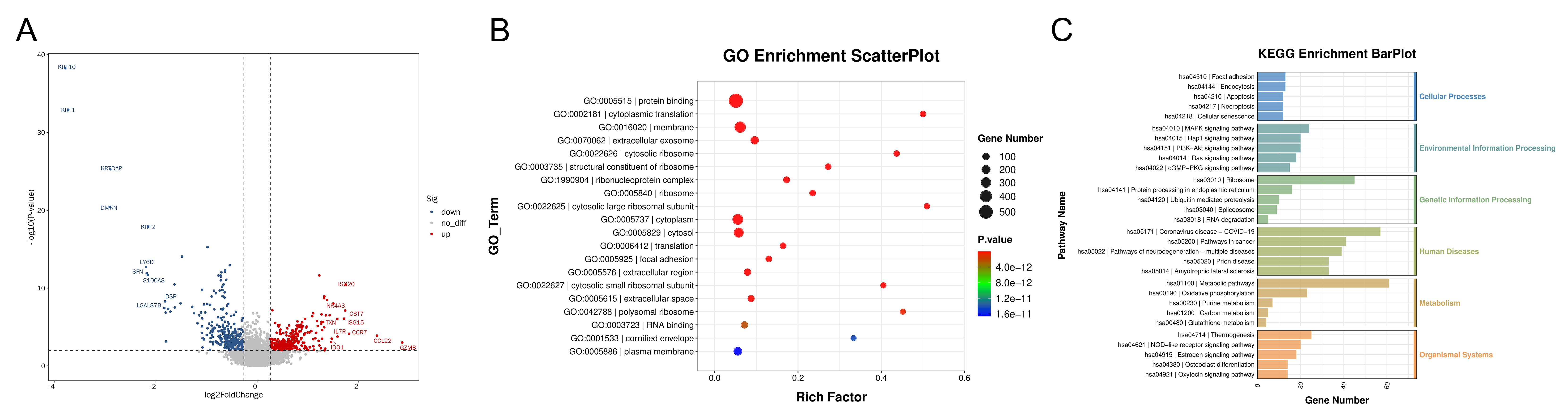

Supplement: SUPPLEMENTARY FIGURE S6 — Identification differentially expressed genes (DEGs) and functional analysis of DEGs in dendritic cells. (A) The heatmap of DEGs in dendritic cells. (B) The gene ontology (GO) analysis of DEGs in dendritic cells. (C) The Kyoto encyclopedia of genes and genomes (KEGG) enrichment of DEGs in dendritic cells. [file Image_6.TIF]

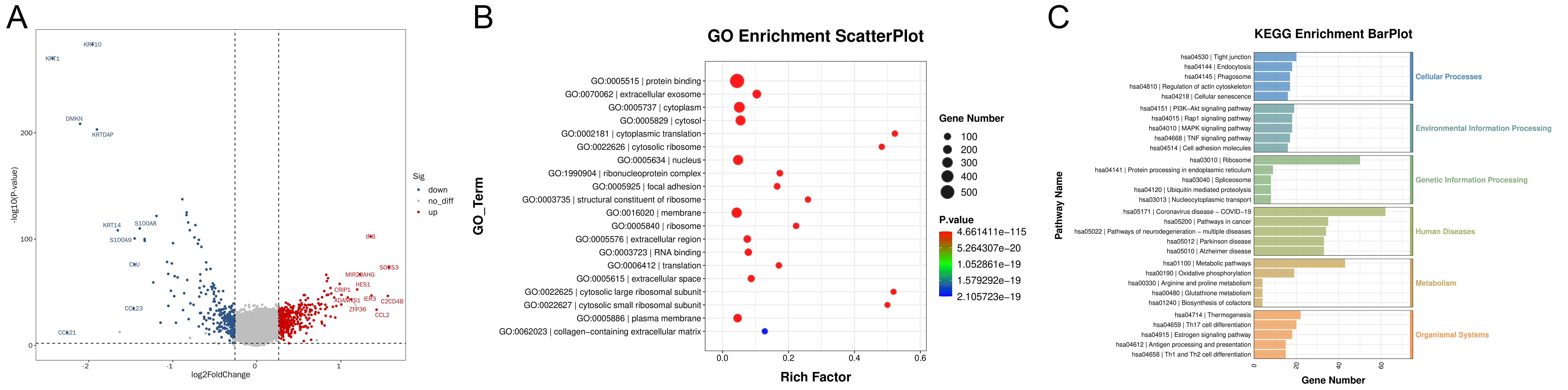

Supplement: SUPPLEMENTARY FIGURE S7 — Identification differentially expressed genes (DEGs) and functional analysis of DEGs in endothelial cells. (A) The heatmap of DEGs in endothelial cells. (B) The gene ontology (GO) analysis of DEGs in endothelial cells. (C) The Kyoto encyclopedia of genes and genomes (KEGG) enrichment of DEGs in endothelial cells. [file Image_7.TIF]

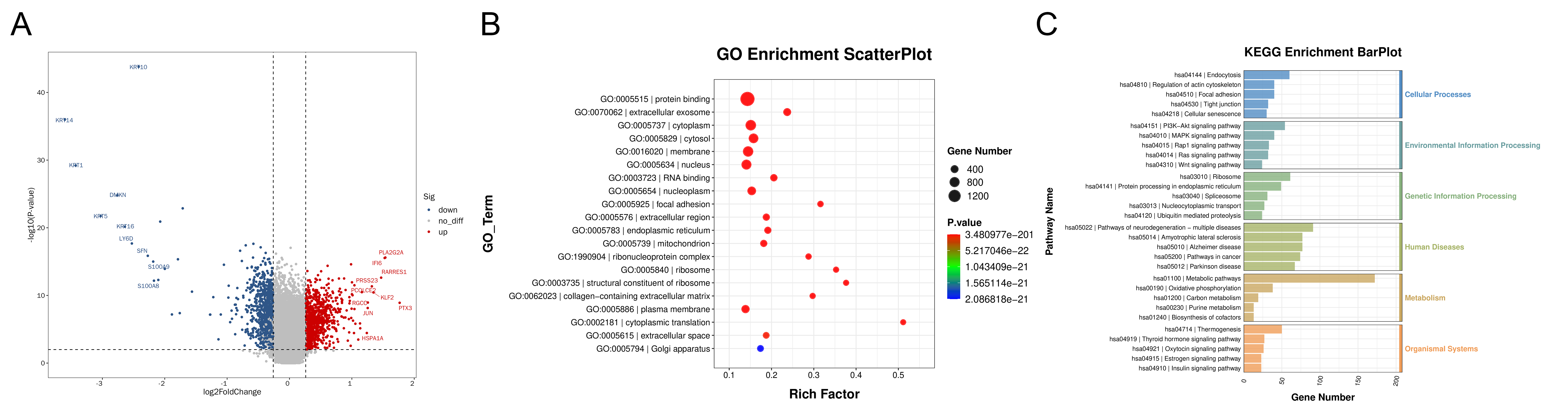

Supplement: SUPPLEMENTARY FIGURE S8 — Identification differentially expressed genes (DEGs) and functional analysis of DEGs in fibroblasts. (A) The heatmap of DEGs in fibroblasts. (B) The gene ontology (GO) analysis of DEGs in fibroblasts. (C) The Kyoto encyclopedia of genes and genomes (KEGG) enrichment of DEGs in fibroblasts. [file Image_8.TIF]

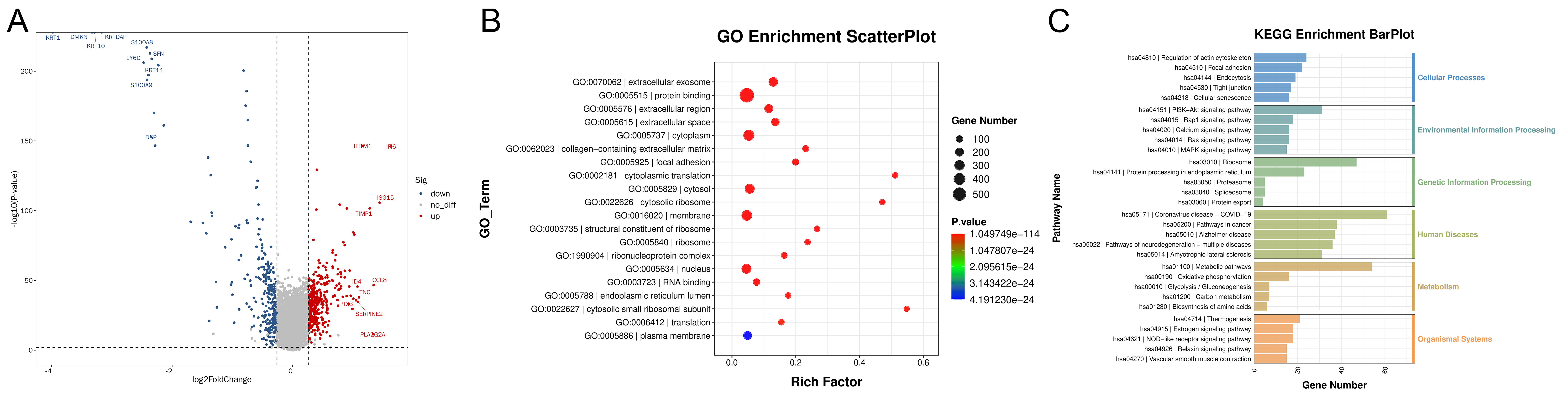

Supplement: SUPPLEMENTARY FIGURE S9 — Identification differentially expressed genes (DEGs) and functional analysis of DEGs in tissue stem cells. (A) The heatmap of DEGs in tissue stem cells. (B) The gene ontology (GO) analysis of DEGs in tissue stem cells. (C) The Kyoto encyclopedia of genes and genomes (KEGG) enrichment of DEGs in tissue stem cells. [file Image_9.TIF]
